# Supplementary material for: A Tentative Study of the Effects of Heat-Inactivation of the Probiotic Strain Shewanella putrefaciens Ppd11 on Senegalese Sole (Solea senegalensis) Intestinal Microbiota and Immune Response
Source: Microorganisms. 2021 Apr 12;9(4):808. doi: 10.3390/microorganisms9040808 (PMC8070671; doi:10.3390/microorganisms9040808)
Supplement: Supplementary file 1 [file microorganisms-09-00808-s001.zip › Figure S1.pdf]

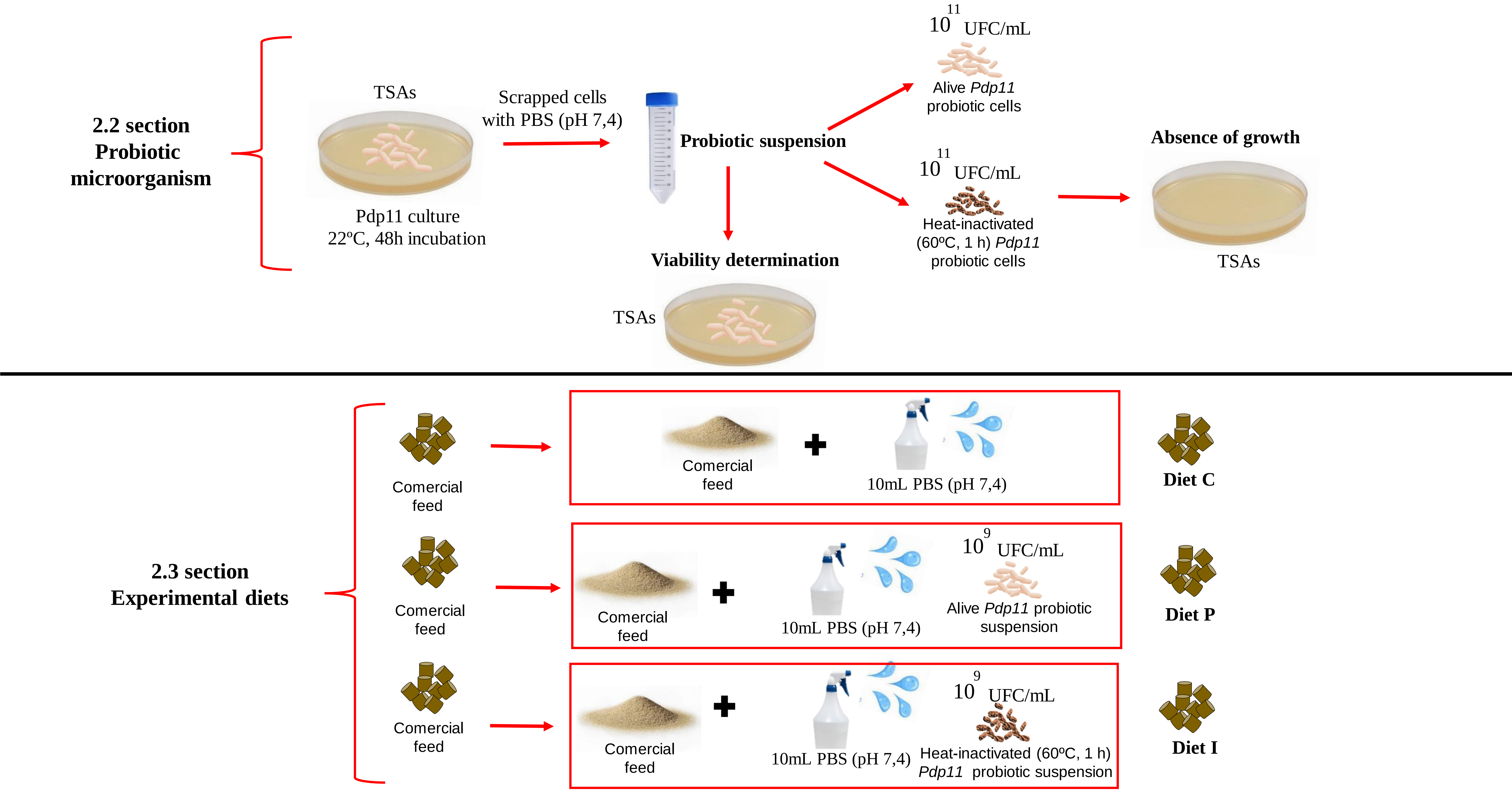

Figure S1. Graphical representation of the sections 2.2 (Probiotic microorganism) and 2.3 (Experimental diets).
