# Supplementary material for: A Tentative Study of the Effects of Heat-Inactivation of the Probiotic Strain Shewanella putrefaciens Ppd11 on Senegalese Sole (Solea senegalensis) Intestinal Microbiota and Immune Response
Source: Microorganisms. 2021 Apr 12;9(4):808. doi: 10.3390/microorganisms9040808 (PMC8070671; doi:10.3390/microorganisms9040808)
Supplement: Supplementary file 1 [file microorganisms-09-00808-s001.zip › Figure S2.pdf]

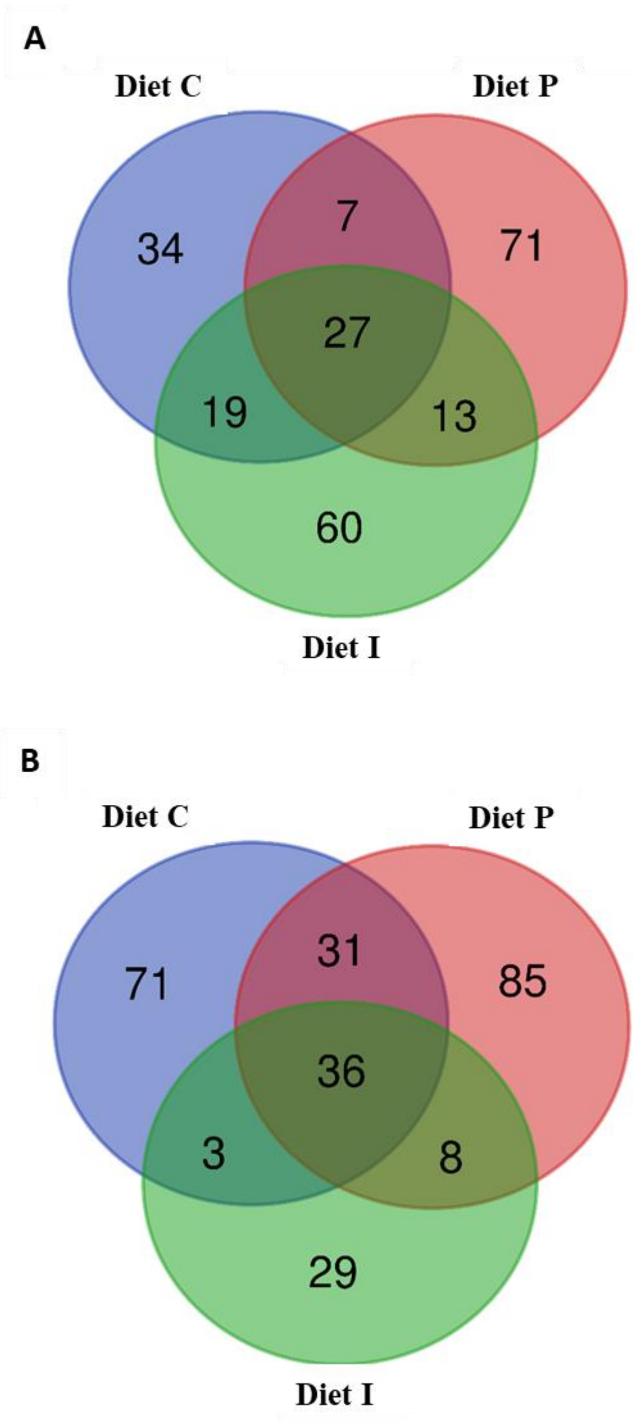

Figure S2. Venn diagrams demonstrating the distribution of genera shared in the anterior (A) and posterior (B) intestinal sections of *S. senegalensis* specimens fed control diet (diet C) and the same supplemented with alive (diet P) or inactivated (diet I) cells of the probiotic. Genera numbers are generated from subsets of each group.
