# Supplementary material for: A Tentative Study of the Effects of Heat-Inactivation of the Probiotic Strain Shewanella putrefaciens Ppd11 on Senegalese Sole (Solea senegalensis) Intestinal Microbiota and Immune Response
Source: Microorganisms. 2021 Apr 12;9(4):808. doi: 10.3390/microorganisms9040808 (PMC8070671; doi:10.3390/microorganisms9040808)
Supplement: Supplementary file 1 [file microorganisms-09-00808-s001.zip › Table S1.pdf]

Table S1. Relative microbial abundances at family level of the anterior and posterior intestinal sections of *S. senegalensis* specimens fed control diet (diet C) and the same supplemented with alive (diet P) or inactivated (diet I) cells of the probiotics Pdp11. Blanks indicate relative abundance percentage below 1%.

**Family**	**Anterior**			**Posterior**		
	Diet C (%)	Diet P (%)	Diet I (%)	Diet C (%)	Diet P (%)	Diet I (%)
Alcaligenaceae		2.48				
Bacteroidaceae		7.89				
Bradyrhizobiaceae		1.19				
Brevinemataceae	13.44	3.34	50.74	77.07	13.61	88.19
Colwelliaceae		1.24				
Enterobacteriaceae		3.34				
Lachnospiraceae		2.49				
Micrococcaceae	1.08					
Moraxellaceae	1.24	1.31				
Muribaculaceae		6.24				
Mycoplasmataceae_f1	67.18	3.80	32.32	7.17	54.33	
Pelagibacteraceae		1.01				
Prevotellaceae		4.04				
Propionibacteriaceae		1.19				
Pseudomonadaceae	3.71	16.41	1.94		2.06	
Rikenellaceae		1.69				
Ruminococcaceae		4.65				
Sphingomonadaceae		1.78				
Spirochaetaceae				9.96	25.78	
Vibrionaceae			4.26	1.68		10.60
Xanthomonadaceae	5.72	20.75	3.59	1.04	1.57	
ETC<1	8.71	15.17	6.07	3.09	2.66	1.21
